# CrowdMed-II: a blockchain-based framework for efficient consent management in health data sharing

**DOI:** 10.1007/s11280-021-00923-1

**Published:** 2022-01-01

**Authors:** Chaochen Hu, Chao Li, Guigang Zhang, Zhiwei Lei, Mira Shah, Yong Zhang, Chunxiao Xing, Jinpeng Jiang, Renyi Bao

**Affiliations:** 1 DCST, BNRist, RIIT, Institute of Internet Industry, Tsinghua University, Beijing, China; 2 BNRist, DCST, RIIT, Institute of Internet Industry, Tsinghua University, Beijing, China; 3 Institute of Automation, Chinese Academy of Sciences, Beijing, China; 4 Joint Research Center for Industry Trust Blockchain Application Technology, Tsinghua University, Beijing, China; 5 Yidu Cloud (Beijing) Technology Co., Ltd., Beijing, China

**Keywords:** Blockchain, Smart contracts, Consent management, Health data

## Abstract

The healthcare industry faces serious problems with health data. Firstly, health data is fragmented and its quality needs to be improved. Data fragmentation means that it is difficult to integrate the patient data stored by multiple health service providers. The quality of these heterogeneous data also needs to be improved for better utilization. Secondly, data sharing among patients, healthcare service providers and medical researchers is inadequate. Thirdly, while sharing health data, patients’ right to privacy must be protected, and patients should have authority over who can access their data. In traditional health data sharing system, because of centralized management, data can easily be stolen, manipulated. These systems also ignore patient’s authority and privacy. Researchers have proposed some blockchain-based health data sharing solutions where blockchain is used for consensus management. Blockchain enables multiple parties who do not fully trust each other to exchange their data. However, the practice of smart contracts supporting these solutions has not been studied in detail. We propose CrowdMed-II, a health data management framework based on blockchain, which could address the above-mentioned problems of health data. We study the design of major smart contracts in our framework and propose two smart contract structures. We also introduce a novel search contract for searching patients in the framework. We evaluate their efficiency based on the execution costs on Ethereum. Our design improves on those previously proposed, lowering the computational costs of the framework. This allows the framework to operate at scale and is more feasible for widespread adoption.

## Introduction

The new coronavirus COVID-19 has a strong transmission and infection capacity [24], and has infected about 229 million people in more than 200 countries or territories. However, the panic caused by the disease’s strong infectivity is more disturbing than the illness itself. This suggests that the timely dissemination of accurate and credible information is essential to counter such threat to public health [23]. There is an urgent need to establish a system for sharing and dispersing medical information so that patients can share their health data with their doctors for remote monitoring, and even with the public. In addition, as mentioned in [1], security vulnerabilities, user data privacy concerns, lack of transparency and other issues will affect the public’s acceptance of such information sharing applications. In order to ensure the public’s acceptance, such a data sharing system needs to ensure the authenticity of the data, that is, the data source is traceable, the transparency of the sharing mechanism, and the security and reliability of the sharing system.

Such a system would also address the problem of health data fragmentation. Patients visit multiple medical institutions throughout their lives, and their medical histories are scattered in these institutions, and neither the doctor nor the patient is able to access an integral medical history [42]. This may have a negative impact on the quality of medical care for patients, as doctors do not have sufficient information to determine their diagnosis and treatment. Moreover, because of the sensitivity of health data, systems that facilitate the sharing of health information must give priority to patients’ right of privacy [5, 9]. That is, patients should be empowered to decide who can access their data and what parts of it they can access.

In such a health data sharing system, because the data comes from different medical institutions all over the world, it is impossible to guarantee the same structure of health data of each institution. Similarly, the quality of data is not guaranteed, so parsing data and cleansing data need a lot of work by people with professional knowledge. This is very unfriendly for medical researchers who use data. On the one hand, it will take up too much of their time. On the other hand, due to interdisciplinary research, they may lack corresponding experience [10]. Therefore, this system needs to improve the quality of data and lower the threshold of using data, which can be achieved by introducing data reviewers into this system.

Besides, patients may lack the motivation to share health data with medical researchers because they do not receive immediate benefits from doing so. Nor can reviewers benefit from reviewing data as well. Therefore, a system that rewards patients for sharing data and reviewers for reviewing data will facilitate the creation of an ecosystem that motivates patients to share their data and reviewers to review data, and the community as a whole will benefit from improved research.

Considering the high cost and complex technical support, traditional centralized health data sharing system mostly adopts cloud-based storage system. However, when they store patients’ health data on cloud servers, they encounter data integrity, authentication, privacy violations, and so on. Because of centralized management, health data can easily be stolen, manipulated, or even totally removed [6, 28]. These systems also ignore patient’s authority and privacy [31]. In addition, due to the resistance between various countries, these centralized data sharing systems are difficult to share data across countries.

A system based on a blockchain may meet these requirements. A blockchain is a tamper-resistant, append-only chain of records stored in nodes in a network. Use public and private key cryptography to maintain consistent transaction records across multiple machines without a trusted third party. As copies of these ledgers are stored on each machine, they are accessible to all members, thus providing auditability and accountability. Using a consensus algorithm, records are packaged into blocks and appended to the blockchain through a process called mining. In Bitcoin’s Proof of Work consensus agreement, miners compete for bookkeeping rights by solving a computationally intensive puzzle. The successful mining node gets bookkeeping rights, add a block (or set of transactions) to the chain, and get some Bitcoin rewards for creating a new block. This new block is broadcasted to other nodes to ensure that all nodes in the Bitcoin network keep consistent transaction records. Mining activities in the blockchain bring the possibility of incentivizing certain behaviors [6].

In order to meet the challenges mentioned above, we propose CrowdMed-II based on CrowdMed we previously proposed [31], a blockchain-based method for managing patient consent and sharing health data. On the basis of CrowdMed, we add the role of data reviewer to the health data sharing system to improve the quality of shared data beyond, not just the sharing of health data. Adding data reviewer to the system to review patients’ health data can improve the quality of the data and help medical researchers make more effective use of these health data. The patient’s medical history data is stored and provided by the institutions that provide medical services to the patient, so that data, in addition to maintaining the most important raw information, implicitly contains knowledge and experience from only one medical service provider. Reviewers’ comments can bring more perspectives. Not only can medical researchers get more information, in addition, the attending doctor can also view the reviewers’ comments to think from multiple perspectives and get further improvement.

The CrowdMed-II can be integrated into the existing data management infrastructure and is very easy to adopt [12]. CrowdMed-II is based on the Ethereum network because Ethereum supports not only mining activities but also smart contracts that meet our design requirements. Smart contract is a kind of computer protocol designed to propagate, verify or execute the contract digitally. In the past research, the design and implementation of smart contracts in health data sharing system has not been discussed and evaluated. In this paper, we study the design of smart contracts to support the large-scale adoption of our proposed framework and design two smart contract structures. These two structures are Patient-Viewer Relationship (PVR)-Centric contract structure and Provider-Patient-Viewer Relationship (PPVR)-Centric contract structure.

Each of the two structures we propose has its advantages and disadvantages, and depending on the circumstances, different frameworks can be chosen. The advantage of the PVR-centric contract structure is that even health data providers can not modify existing data in the smart contract. Although this is not enough to guarantee that the data in providers’ local databases will not be tampered, tampering can be detected. The disadvantages of the PVR-centric contract structure are that it takes up more storage space and doesn’t support correcting data. Each time providers append data to their local databases for the same patient, they can only add records in the smart contract. This will take up a large amount of storage space, and the storage space on the blockchain is very valuable. The PVR-centric contract structure does not support correcting data, which puts forward high requirements for health data providers. Maybe it will reduce their enthusiasm. Even if the imported reviewer can make some adjustments, the process is still tortuous and unfriendly to the data viewer. The advantage of the PPVR-centric contract structure is that it supports updating. On the one hand, when data is appended to a local database, the health data provider can simply update old records without creating new records in the smart contract, saving valuable space. On the other hand, health data providers can create data more freely because they can correct erroneous data. Although the provider can change the data in smart contracts, these changes are made through sending transactions, so they can be backtracked through transactions. While this can be cumbersome, it can also support obtaining the modification history of the provider. In addition, it makes sense that providers can view and manage the data they provide. The disadvantage is that the data modified by providers is not very friendly to researchers. This may make the data used by researchers unable to reproduce, which requires researchers to store the data they used locally.

In addition, we propose group-based access rights to improve the efficiency of storing and updating access rights on the hypothesis that a user is likely to define access groups for groups of viewers rather than unique permissions for individual users. We have also proposed a new contract to support the search for patient data that will enable medical researchers to discover who has appropriate data to meet their needs. This is a novel feature in health data sharing systems and this feature would be useful to medical researchers looking to obtain patient data. These two smart contract improvements can be applied to both of the above two smart contract structures and bring enhancement.

As the number of users and network density increase, the computational costs of executing these smart contracts may rise to the point where the framework can no longer run. Through simulation and experiment, it is shown that our smart contract designs are much improved than these previous designs. Our design has lower computational costs and is extended sustainably as the network grows.

Part of the above work has been explored to a certain extent in our previous paper [32]. In that paper, we constructed PVR-Centric contract structure based on CrowdMed, proposed search smart contract and group-based access rights, and carried out experiments on them. In this paper, we expand that paper in the following three aspects. Firstly, we propose a novel framework CrowdMed-II. By introducing the role of data reviewer, the previous framework CrowdMed has been improved. CrowdMed-II can improve the quality of data shared in the system and promote the sharing and utilization of data. Secondly, we propose a novel smart contract structure PPVR-Centric contract structure in the consideration of the data creator’s need to update the records. PPVR-Centric contract structure supports update records, which can save valuable storage space of the blockchain and also provide convenience for data creators. Lastly, we conduct experiments on the novel framework and smart contract structure. Because of the use of CrowdMed-II, not only the new smart contract structure, but also the previous smart contract structure, must be experimented based on the new framework. Through these experiments, we validate our design.

The main contributions of this paper are summarized as follows:

1. We propose CrowdMed-II, a new health data management framework based on blockchain, which could address the problems of data fragmentation, data quality, data sharing and patient privacy in health data.
2. We propose group-based access rights to improve the efficiency of storing and updating access rights. We also design a novel search contract that supports searching patient data. This is a new feature in health data sharing systems. We implement these two smart contract improvements and assess the practicability of our design.
3. We design two smart contract structures and implement them as well as another proposed previously [6]. With the implementation of group-based access rights to these three structures, we get six smart contract systems and evaluate them. We demonstrate that our proposed smart contract structures and improvement scheme are better than those previously proposed.
4. We deploy these smart contracts into an Ethereum test network and conduct extensive simulations to evaluate these smart contracts and show the benefits of the improvement we proposed. To the best of our knowledge, this is the first in-depth study in the development of smart contracts for health data sharing where contracts were evaluated in isolation.

## Related work

In this section, we give an overview of research that has been done in health data sharing. First, we review some proposed systems that integrate heterogeneous health data using traditional database management technologies. Next, we describe the use of blockchain in health data sharing and some blockchain-based systems that others have proposed. Last, we introduce blockchain technologies and smart contracts that support our framework.

### Traditional health data sharing system

By using the traditional database storage system, Khennou et al. [21] propose using the mobile agent paradigm to transfer health data from multiple databases to a centralized database. Gui et al. [17] propose a healthcare big data management and analysis architecture focusing on data pre-processing and data transforming. Due to the high cost and complex technical support, these systems usually use cloud-based storage system to store health data centrally. Thus, they encounter a variety of security threats, such as data theft, data corruption, data integrity, authentication and privacy violations [6]. As for patient’s authority and privacy, Riad et al. [29] proposed an access control mechanism (SE-AC) for cloud-based IoT healthcare systems, which enables patients to control their own health data. However, these measures are located in the cloud environments to obtain security, so there are still challenges. In addition, due to the resistance between various countries, these centralized data sharing systems are difficult to share data across countries.

Blockchain provides a new way to solve the inherent security challenges in cloud-based systems. Therefore, the development of blockchain-based medical systems has been paid more attention [20].

### Blockchain in health data sharing

The authors of [2, 6, 15, 25, 33] propose blockchain-based systems for sharing health data securely, which use smart contracts and permission contracts to protect patients’ authority and privacy. However, they either do not solve the problem of data sharing between medical institutions, or utilize a centralized database for data storage, or do not fully protect the controllability of patients. Kumar et al. [22] outlined potential applications of the blockchain in the area of healthcare, including data sharing, data access control and medical history maintenance. The authors emphasize using smart contracts to implement the practical application of blockchains and raise concerns about scalability when developing such applications. We solve this problem directly by studying the scalability of smart contracts developed for health data sharing.

Several blockchain-based systems have been proposed for the secure sharing and transmission of health data, such as [2, 6, 7, 11, 33]. In our research, smart contracts are the crucial innovation in achieving these goals. However, the authors of [2, 7] do not go into the design of smart contracts at length. The authors of [33] provide a brief overview of the smart contracts implemented in their systems, while [6, 11] detailing the structure and functionality of their proposed smart contracts at the same time. However, neither paper implements nor conducts simulations on smart contracts individually. In this paper, we evaluate the proposed smart contract design and compare it with our novel design.

In [6], Asaph et al proposed the Patient-Provider Relationship (PPR)-Centric contract structure. PPR-Centric contract structure uses the three types of contracts presented in [6]. The Registrar Contract (RC) stores the mapping of a user’s unique identifier to his or her Ethereum address and the Summary contract (SC). The patient’s SC contains information about reconstructing patient history as well as most of the critical functions of the blockchain. It stores a list of PPR contracts, as well as a status object that informs the user of changes to his or her PPR contract. PPR contracts between patients and medical service providers store information on the patients’ medical records created by the medical service provider. The creation of medical records, viewer authorization, and data requests are sent directly to SC, which updates the relevant PPR contract accordingly or retrieves the requested query string from the related PPR contract. PPR-Centric contract structure supports data sharing, but only by integrating the data across multiple PPR contracts can we get the complete data of a patient. In this paper, we propose two new smart contract structures.

Besides, in our literature review, the ability to search patients’ data on the blockchain is not explored.

### Blockchain technologies and smart contract

The success of Bitcoin has stimulated interest in studying other applications of the blockchain [26]. This paper is implemented in the Ethereum Network, which introduces smart contracts in the blockchain network. Harris [18] discusses the development of smart contracts and how to avoid common mistakes. These papers help us to develop smart contracts for our framework.

Data provenance is closely related to the function of the blockchain in our framework. An et al. [3] explore smart contracts for data exchange. Neisse et al. [27] propose smart contracts to track data provenance. While these ideas put forward by these authors are applied to different areas, they have inspired our experimentation and design, particularly in evaluating smart contracts. Ruan et al. [30] who focused on the entire system rather than smart contracts have proposed LineageChain, a blockchain system for data provenance tracking. The authors of [37] also developed an access control system in the blockchain which includes a search mechanism for health data. Using a similar approach, we propose a similar system designed for health data sharing.

The search mechanism on the blockchain is a critical part of our research. The authors of [14] have explored how to use smart contracts to securely search for encrypted data stored in a blockchain. Chen et al. [8] proposed a searchable encryption scheme for electronic health record sharing. These ideas presented in these papers inform the design of the search contract we proposed, enabling us to integrate patient data search into a health data sharing system, a new feature that has not been explored before.

Many blockchain platforms are supporting smart contracts, including the famous ETH, RSK and Zen. Ethereum is Turing’s complete and has probably the largest developer community. It supports smart contracts very well. So we implement our framework based on Ethereum.

Ethereum is developed as a generalized application of blockchain. Any transaction-based state machine can be built on the Ethereum basis. This is achieved thanks to the use of smart contracts. Smart contracts are codes stored in a blockchain, and they contain data and functions used to process data. Thus, a smart contract defines a transaction-based state machine. The state of the state machine is stored in the smart contract and can be changed by executing functions in the smart contract. Users execute functions in smart contracts by sending transactions. Transactions sent to the blockchain will be logged, providing auditability.

The smart contracts in Ethereum are written with Solidity, a Turing-complete programming language. In order to prevent the network from being abused and to ensure that all transactions are eventually closed, computation in Ethereum is constrained by gas. Gas is the computational cost in Ethereum, where the user pays the miners a certain amount of gas for each transaction sent to the contract. Each transaction requires the user to specify a gas limit, beyond which transactions will stop being mined and not be added to the blockchain. Therefore, it should be of concern to developers to ensure that the computational costs associated with their smart contracts are not too high and do not increase exponentially as the number of users (and the amount of data stored) increases. As a result, we use gas consumption and scalability to evaluate our smart contracts.

## Framework design

In this section, we give a brief introduction to the users and framework of CrowdMed-II with the addition of a data reviewer role, with a focus on the ability to interact with blockchain components. These provide information for the design of the smart contract in Section 4, and the experiments evaluating smart contracts in Section 5.

**Fig. 1:**
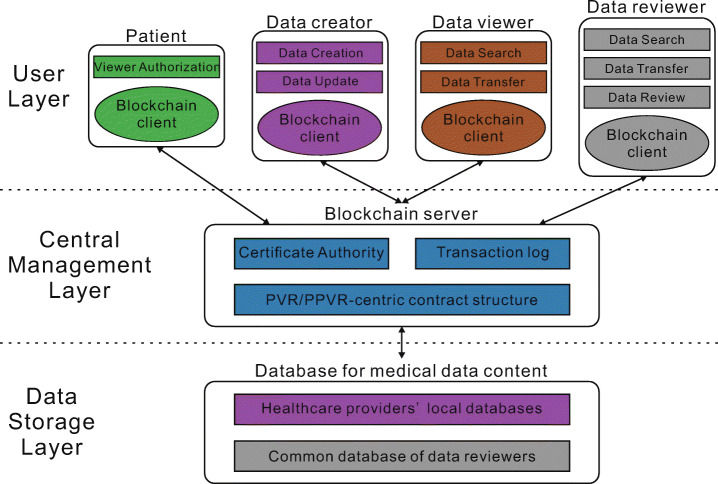
CrowdMed-II architecture

Figure 1 presents the architecture of the CrowdMed-II framework. It is divided into 3 layers: the user layer, the central management layer and the data storage layer. The four types of users in the user layer are described in detail in Section 3.1 and the service functions they use are described at length in Section 3.2. The central management layer is roughly the same as it in CrowdMed. The certificate authority is responsible for verifying the user’s identity and links their real-world identity to a unique virtual ID. This is the signature used by the user in transactions on the blockchain. The blockchain can choose the PVR-centric contract structure or the PPVR-centric contract structure depending on the circumstances. The details of these two smart contract structures are described in Sections 4.1 and 4.2. As for the data storage layer, data creators only upload query strings and data hashes to the health data sharing system rather than the raw health data. These data are stored in their local databases. Comments of data reviewers are also stored in a common database for data reviewers. The framework connects these databases to the blockchain and executes query strings on them.

### Users of CrowdMed-II

The CrowdMed we proposed in [31] divides users into three types: patients, data creators and data viewer. In this paper, we add data reviewers to CrowdMed-II to improve the quality of shared data, not just the sharing of health data.

Patients are the main body of health data and have the core authority over their data. So patients should be the only ones with the right to decide who can access their data, and what part of it can be accessed. In CrowdMed-II, the patient’s primary responsibility is authorization management. They can set different accessibility fields and time frames for different data viewers and different groups. Of course, patients should be able to view their own complete health data without the need for permission from others. In the future, the framework could simply be expanded to include patients as data creators who can upload data to the system, such as monitoring data from a variety of wearable devices [19].

Data creators, primarily medical service providers, are responsible for providing health data and creating corresponding query strings into the system. In fact, when providing medical service to patients, medical service providers usually already create raw health data in their local databases. In this blockchain-based health data sharing system, creating data is actually adding query strings and data hashes corresponding to data in the local database of the medical service provider. Each query string created by a data creator can be traced back to that user, so the data creator is also responsible for the accuracy of the query string. In addition, the data creator needs to maintain the query string. When a patient’s data from the local database is appended, the data creator also needs to supplement or update the query string and data hash in the health data sharing system. In this paper, we regard medical service providers as the only producers of raw health data for the sake of simplicity. In the following, we usually use the medical service provider to represent the data creator.

Data reviewers, similar to, but distinct from, data creators, can be considered as a special class of data creator. The data reviewers do not provide clinical data, but rather comments left after a review of the case information. Since not every data reviewer can have his or her own local database and the amount of data reviewers’ comments is not particularly large, the health data sharing system provides a database for all data reviewers. The data reviewer simply creates the corresponding query string and hash in the system like data creators. Data reviewer must be an authority in the relevant medical field, such as doctors or medical researchers, and the comments created by data reviewers should contain their signatures and will be open to all data viewers. In addition, data reviewers’ comments need to be desensitized, that is, patients’ privacy should not be revealed.

Data viewers are those who request access to patients’ data, primarily doctors and medical researchers. To get a better understanding of the patient’s condition, doctors may request to view more complete historical data of the patient. When medical researchers conduct medical research, they usually need a large number of data samples of the corresponding diseases. In the system, the viewer role is very simple, doesn’t make any changes to the system or provide any services, just gets data.

### Service functions on the blockchain

CrowdMed-II provides service functions for users and users call these functions to realize the corresponding purpose. Actions such as creating data, updating data, sharing data, reviewing data and viewing data are performed by sending transactions to functions in smart contracts, thus creating an immutable log that can be tracked and verified.

The service functions carried out on the blockchain are:

1. Data creation: DC(), which is performed by the data creator (usually a medical service provider) who stores pointers (to the data on the creator’s database) as an entry in the patient’s medical records. In this system, we view this pointer as a query string that can be executed on the creator’s SQL database. The requesting party can then obtain the query string through the blockchain system and execute it on the creator’s database to obtain patient medical records.
2. Data update: DU(), which is performed by the data creator. It is necessary to update the data. Once the data creator appends data in the local database, even if the query string does not need to be changed, the hash of the resulting data is highly likely to change. If the entry in the blockchain is not updated, the query string and the data hash will not match and an error will occur.
3. Data review: DR(), which is performed by data reviewers, creates a comment record that is attached to the original health data being reviewed. The record may contain a variety of data reviewer opinions on the patient’s medical record, such as opinions about the condition or opinions regarding the treatment plan of the medical service provider. In this system, the details of the comment are stored in a database specifically for data reviewers, and the blockchain stores a query string to obtain the data in the database and a hash value for cross-checking.
4. Viewer authorization: VA(), which is performed by the patient and specifies the field or span of data that a viewer can access. In this system, we treat this authorization as a composite query string corresponding to the patient’s medical history that the viewer is allowed to access. This function involves storing query strings associated with a specific viewer in the patient’s permission settings.
5. Data search: DS(), which is performed by a viewer searching patient who matches certain criteria. This function is very necessary, otherwise, the practicability of this health data-sharing system will be greatly reduced. Only with this function can data viewers obtain the data they need directionally.
6. Data transfer: DT(), which is performed by a viewer requesting patient’s data. The contract returns the query string corresponding to the medical record that the viewer is allowed to access, as well as the query string corresponding to the result of data reviewer’s review of the health data.

The smart contracts we proposed are evaluated in this way, comparing the computational cost of performing the above functions with that of the other smart contracts proposed previously.

### Incentive mechanism

Patients maintain an access permission setting for data reviewers and data viewers. Medical service providers create data in the system. Data reviewers review patients’ health data and give comments. Data viewers can use a separate search contract to search for patients who meet their criteria.

In this system, data viewers do not provide any services. They are pure user rather than supporters, and direct beneficiaries. So for data viewers, without any incentive, will actively use the system to obtain the data in their researches. In order to prevent data viewers from having unrestricted access to data, and to make the system run more healthily, data viewers should pay the corresponding fees for obtaining the health data.

Medical service providers bear the heaviest responsibility in the system, not only for creating and updating data in time, but also for the accuracy of the data and for providing access to their local databases. With so much responsibility on the part of such providers, pure token incentives are hard to attract. A wide range of medical service providers can be the co-sponsor of the health data sharing system. As initiators, they can enjoy certain privileges. For example, their medical institutions can have a certain number of viewers to obtain the data shared in the system for free based on their contributions. In this way, these institutions providing medical services can also derive sufficient benefits from the system.

Although data reviewers are also a special kind of data creator, they cannot be treated in the same way. If we just give data reviewers free access, on the one hand, it can not encourage data reviewers to review more health data and give more serious comments. On the other hand, data viewers may register data reviewer accounts to obtain research data for free without review. But data reviewers can only review and comment on the data once they get it. So, in this health data sharing system, data reviewers have to pay the same fees as the data viewer to get the data. But after data reviewers review and comment, the system eventually gives data reviewers enough tokens to cover the expenses of viewing the data, and there is still plenty of surpluses. This also motivates data reviewers to conduct more reviews and provide high-quality comments.

Finally, the system is sharing the health data of patients, but patients do not benefit directly from this sharing. To encourage patients to authorize more viewers to view their data, the system gives patients a one-time basic reward for opening data and a continuing incentive for the patients’ data to be viewed every time. These two types of rewards not only encourage patients to open data but also offer more rewards and fairness to patients who share more data.

The fees or rewards here are issued and used in the form of tokens in the system. The tokens should be able to be exchanged for real-world benefits, as determined by the government. In this way, patients, medical service providers, data reviewers and viewers are able to derive sufficient benefits from such a system to motivate them to contribute to it and thus become a sustainable ecosystem.

## Smart contract design

In this section, we introduce the implemented smart contracts in detail. Sections 4.1 and 4.2 introduce two different smart contract structures we proposed that support the service functions described in Section 3.2. Section 4.3 introduces two improvements that can be applied to these structures.

### Patient-viewer relationship-centric contract structure

**Fig. 2:**
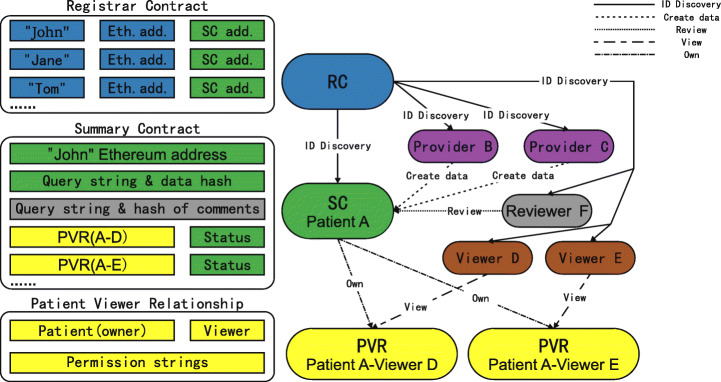
PVR-centric contract structure

In the Patient-Viewer Relationship (PVR)-Centric contract structure, we use three types of contracts, as shown in the Figure 2. RC is the same as RC used in the PPR-Centric contract structure. It stores the mapping of a user’s unique identifier to his or her Ethereum address. This contract is used to associate users’ real-world identities with their digital identities. The unique identifier is a hash of the real-world identifier.

In this structure, the patient’s SC itself contains complete patient medical records, complete data reviewer commentary information, and the critical functions of the blockchain. Patients’ medical records are stored in smart contracts as a list of database query strings that can be executed on providers’ databases to recover the medical records themselves. To ensure data integrity, a list of hash values of data are also stored in the contract for cross-checking. Similarly, the original content of the data reviewer’s comment is stored in a database provided specifically for data reviewers, and then in the corresponding PPR contract as a string of database query, with a hash value for cross-checking. SC also stores a list of PVR contracts, as well as status objects that notify users that changes have occurred to their PVR contracts. The creation of medical records and data reviewer comments are directed to and processed by SC. The viewer authorization and data request are directed to SC, which then updates the corresponding PVR contract or retrieves the query string accordingly.

A PVR contract between the patient and the viewer deals with the details of the viewer’s permission. The PVR contract stores the query strings corresponding to a subset of the medical records stored in SC and these query strings are managed by the patient. When the viewer requests patients’ data, the viewer sends the transaction to SC. Using the sender’s identity details, the SC finds the appropriate PVR contract and returns the query string stored in the PVR to the viewer.

We propose this contract structure because it supports more efficient retrieval of medical records as they are stored in a single SC contract, rather than across multiple PPR contracts. Once permissions are set, the query string also only needs to be retrieved in a PVR contract, unlike the PPR structure, which requires multiple PPR contracts to integrate. In the case of patients interacting with many medical service providers, that is to say, when there are many PPR contracts belong to one patient, the method of iterating over various PPR contracts to obtain the complete medical records of patients significantly increases the computational cost in the blockchain. Therefore, we can hypothesize that the PVR-centric contract structure will be more scalable with the increase of the number of patient-provider relationships.

### Provider-patient-viewer relationship-centric contract structure

**Fig. 3:**
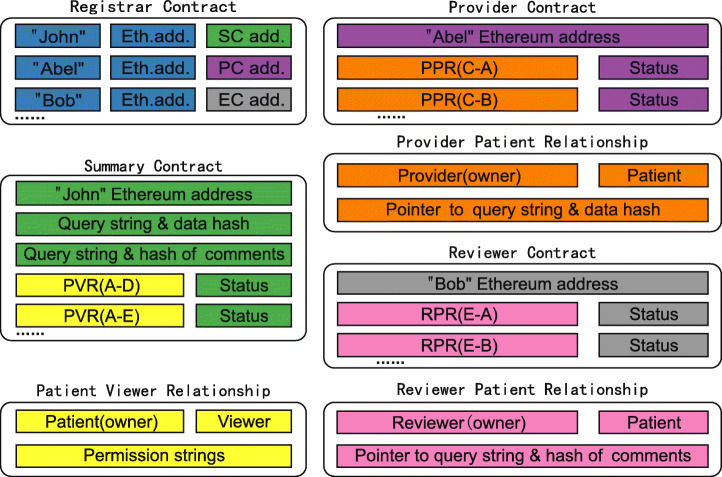
Data stored in PPVR-centric contract structure

**Fig. 4:**
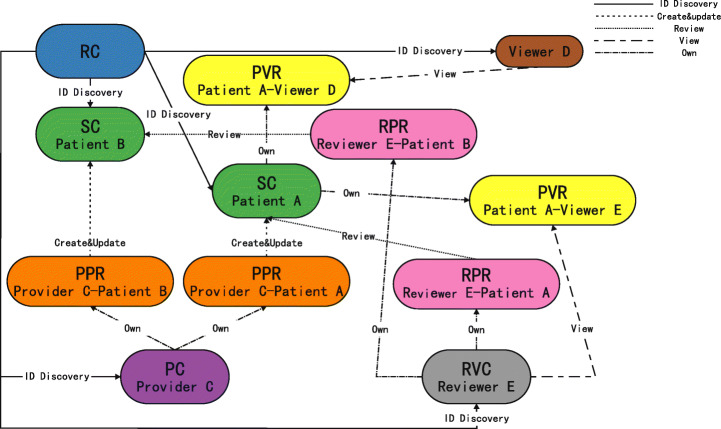
Relationship between contracts in PPVR-centric contract structure

Based on PPR-centric constact sturcture and PVR-centric contract structure, and considering their respective advantages and disadvantages, we further propose the contract structure with Provider-Patient-Viewer Relationship (PPVR) as the center. In addition to making the appropriate modifications to the RC and SC used in PPR-centric contract structure and PVR-centric contract structure, we have added two smart contracts: the Provider Contract (PC) and the ReViewer Contract (RVC). The data stored in each contract is illustrated in Figure 3 and relationships between these contracts are illustrated in Figure 4.

The RC in the PPVR-centric contract structure is logically similar to the previous RC, but not identical. In the PPVR-centric contract structure, patients, medical service providers, data reviewers and viewers are more distinct: For a patient, RC stores a mapping of the user’s unique identifier to his or her Ethereum address and the SC; For a medical service provider, RC stores a mapping of the user’s unique identifier to his or her Ethereum address and PC; For a data reviewer, RC stores a mapping of the user’s unique identifier to his or her Ethereum address and EC; For a viewer, he or she doesn’t have personal smart contract, so RC stores only a mapping of the user’s unique identifier to his or her Ethereum address. The boundaries of the four roles are very clear, with different registration processes depending on the type of registered user.

The SC follows the SC in PVR-centric contract structure, the stored data is unchanged, and the functions are similar. In PVR-centric contract structure, SC deals with most of the functions in the blockchain, including creating medical records, viewer authorization, and so on. In the PPVR-centric contract structure, the functions performed by medical service providers are isolated and placed into the PC. Similarly, the functions performed by data reviewers are also separated into EC. Of course, since creating data, updating data and review are essentially manipulating the data stored in SC, only the external access interfaces of these functions are placed in the PC and EC. In fact, the actual execution process is still in SC, but it is not externally visible, called indirectly through external interfaces in PC and EC. This design makes the functions of each contract more clearly defined. In general, creating data, updating data and review are indirectly changing the data stored in the corresponding SC through PC or EC, while authorization management and data requests are directed to the SC, modifying or reading the patient-viewer relationship.

PVR does not appear as a separate contract in the blockchain, but as just a relationship stored in SC. This relationship naturally implies the identity of the patient, and it also needs to explicitly include the viewer’s identity information and permission strings. The data structure of PVR should support the use of the viewer’s identity information to quickly locate the corresponding relationship, which can be done through mapping. This design, which does not treat PVR as a separate smart contract but as an item in the SC, saves resources in blockchain and facilitates patients to manage authorization and viewers to request data.

The PC belongs to a medical service provider and contains all patient information provided by the provider. Providers create and update data through PC. To create data, the provider adds a provider-patient relationship record to the PC, and then indirectly add the query string and data hash in the corresponding patient’s SC. When the data update belongs to this provider-patient pair is updated (the increase in data in the provider’s local database corresponds to data updates in the blockchain), the provider can update the query string and data hash in the SC of the corresponding patient through the existing provider-patient relationship.

The provider-patient relationship is quite different from the patient-provider relationship in the PPR-centric contract structure. First, the owner of the provider-patient relationship is the provider, not the patient, which is a fundamental difference. Second, the provider-patient relationship is just a relationship, not a contract, stored as an entry in the provider’s PC that saves resources on the blockchain and is more efficient. Finally, the functions are different. In the PPR-centric contract structure, PPR contract stores data, and only by integrating the data across multiple PPR contracts can we get the complete data of a patient. In PPVR-centric contract structure, the provider-patient relationship is for the provider to manage all the patients he is responsible for, and doesn’t need to store the patient data.

The RVC is designed in exactly the same way as the PC, and the data reviewer can be seen as a special provider, except that the data provided by the data reviewer is not clinical data, but comments made after reviewing the clinical data. Unlike each provider has its own local database, data reviewers’ comments are stored in a database specially provided by the health data sharing system for data reviewers. Then data reviewers are just like providers. The SC stores query string and data hash, and the RVC stores information about all patients reviewed by the data reviewer. The reviewer-patient relationship (RPR) is also similar to the provider-patient relationship.

We propose the PPVR-centric contract structure for two reasons: On the one hand, it is very practical for providers and data reviewers to manage the information of those patients they are responsible for; On the other hand, there is a real need to update data in such a health data sharing system. When appending data to the provider’s local database, if a new query string and a data hash must be created in the blockchain for each additional local data, it will greatly waste the valuable storage resources in the blockchain. This will also increase the complexity of the data, make it difficult for patients to manage the authorization and the viewer to find the data, and reduce the efficiency of the system.

Adding the role of data reviewer to the PPVR-centric contract structure is intended to improve the quality of data and allow medical researchers to make more effective use of the data provided by such a health data sharing system. In addition, medical service providers can gain further improvements by viewing data reviewers’ comments and communicating with a large number of doctors or academics in the same field, which can also be beneficial for patients and lead to better medical care.

With the design of the PPVR-centric contract structure above, we can hypothesize that the PPVR-centric contract structure is even more scalable than the PVR-centric contract structure as the number of patient-provider relationships increases. Not only can we efficiently manage and retrieve data, but also can update data efficiently.

### Group-based access rights and search contract

We assume that users may define access groups for viewers and authorize groups, rather than defining unique permissions for individual users. For example, the patient may authorize all doctors who interact with him or her to have access to his or her complete health data, and all data reviewers and medical researchers to be granted a subset of his or her full complete health data. Therefore, we propose group-based access rights to improve user convenience and the efficiency of our smart contracts. Patients defined several groups of viewers, such as “hospital”,“ data reviewers “and” researchers ”, and assigned access rights to these groups. Then, when a new viewer requests access to a patient’s data, the patient only needs to simply assign the viewer to a preset group. As shown in the Table 1, when there are only six users and they belong to three groups, the individual-based access authorization smart contract needs to maintain six permission strings, while the group-based access authorization smart contract only needs to maintain three. With the growth of the number of users, the gap is growing.

**Table 1:** Individual-Based Access Rights VS Group-Based Access Rights

Viewers	Authorization for individuals	Authorization for groups
Doctor A	Permission string for A	Permission string for doctor group
Doctor B	Permission string for B	
- - - - - - - - - - - - - - - - - - - - - -	- - - - - - - - - - - - - - - - - - - - - - - - - -	- - - - - - - - - - - - - - - - - - - - - - - - - -
Reviewer C	Permission string for C	Permission string for reviewer group
Reviewer D	Permission string for D	
- - - - - - - - - - - - - - - - - - - - - -	- - - - - - - - - - - - - - - - - - - - - - - - - -	- - - - - - - - - - - - - - - - - - - - - - - - - -
Researcher E	Permission string for E	Permission string for researcher group
Researcher F	Permission string for F	
- - - - - - - - - - - - - - - - - - - - - -	- - - - - - - - - - - - - - - - - - - - - - - - - -	- - - - - - - - - - - - - - - - - - - - - - - - - -
sum	6	3

This modification to the smart contract improves the efficiency of storing and updating access rights. First, instead of storing multiple copies of complex query strings for each viewer, we simply store the query string once per group and map the viewer to the group. Second, updating the searcher’s query string (for example, when a new medical record is created) is very effective, because a group has only one of the query strings to update, rather than updating it for each member of that group over and over again. As a result, access rights are easier to maintain. Our framework does not limit the number of groups that a patient can create, but a viewer can only belong to one group, so the normal group size is not greater than the viewer size. Thus, while making access easier to maintain, patient delegation flexibility has not been sacrificed. If patients need it, they can still set specific access rights to a single person.

As mentioned above, without the search function, the practicability of this health data sharing system will be greatly reduced. Only with this function can medical researchers obtain the data they need directionally.

In order to realize the search function of our framework, we adapt the ideas presented in [14]. The search contract maintains the mapping of keywords to patients and returns a list of patients that matches the input keyword. These keywords can be updated by patients, doctors, and data reviewers, who not only add missing ones but also review the accuracy of existing ones. For example, patients can disclose their personal information, such as age and sex, to searchers by attaching the keyword “gender: man” to their keyword list. At the same time, doctors can add keywords such as “heart disease” or other terms that describe disease and prescription to the patient’s keyword list. The purpose of adding keywords to patients’ health data is to better serve data viewers and enable them to obtain target data efficiently, and thus promote data sharing. These keywords are just general information like gender, age, disease type, etc., which do not involve the specific identity of patient. We can not lock the real identity of patient through these keywords, nor can we get the specific condition of the patient. Therefore, we store keywords in the smart contract in plaintext, and use hash table as the storage structure to achieve efficient retrieval. When a query is executed, the query entry is hashed, and then the matching patient is quickly determined. Once the patient address list is available, searchers can request access to patient data using the function provided by the smart contract above.

To limit the computational of operations that should cost as little as possible, keyword searches are performed on a single query term. The client then processes the ranking of returned patients based on how well they match multiple query terms.

These two improvements can be applied to the above three smart contract structures. We implement them and assess the practicability.

## Experiments

In this section, we describe the experiments conducted to evaluate the smart contracts described in Section 4, and present the results of the experiments. PPR-Centric contract structure proposed in [6] is set as our baseline and we compare three different smart contract structures. We run two different experiments. The first experiment evaluated smart contract structures based on gas consumption. Through this experiment, we select the most optimal structure to conduct the second experiment and carry out further performance evaluation to measure transaction throughput and latency.

### Experimental data

For our experiments, we use electronic medical records from the publicly available MIMIC-III critical care database. MIMIC-III is a large, freely-available database comprising de-identified health-related data associated with over forty thousand patients who stayed in critical care units of the Beth Israel Deaconess Medical Center between 2001 and 2012. MIMIC-III contains many tables of data, such as ADMISSIONS, CALLOUT, ICUSTAYS, CAREGIVERS, and so on. But as mentioned above, we store the original health data in the local database of the medical service provider, and our smart contract stores query strings and data hashes corresponding to that medical records. Thus, We first generate query strings that retrieve complete medical records from the local database corresponding to a patient visiting a hospital. The SHA-256 hash of this medical record is then obtained. Finally, we get enough query strings and data hash pairs of health data, which is the experimental data we eventually use in the blockchain. These query string and data hash pairs are then randomly drawn to simulate the event where a patient visits a medical provider. In this experiment, we don’t use all the data tables, but mainly use three tables: the admissions table, the patients table, and the prescriptions table. The statistics of these tables are shown in the Table 2.

**Table 2:** The statistics of three tables in MIMIC-III

Table	Number of rows
admissions	58,976
patients	46,520
prescriptions	4,156,450

### Experimental setup

For our first experiment, we evaluate our smart contracts by executing a sequence of transactions on a simulated Ethereum network, logging the gas consumption of each transaction. The simulated network uses ganache-cli v6.5.0. Smart contracts were written in Solidity v0.5.13 and compiled using solc v0.5.10. For the second experiment, we executed the same sequence of transactions on a locally hosted Ethereum network, measuring transaction throughput and latency. The network was built using geth v1.9.12.

### Experimental design

We have implemented the PPR-centric contract structure proposed in [6], as well as our PVR-centric contract structure and PPVR-centric contract structure. We apply an additional improvement, the group-based access authorization, to these three smart contract structures. So we finally get six smart contract systems and conduct the following two evaluation experiments. The test network configuration of the two experiments is consistent, including 10 medical service providers and 500 patients.

For the first experiment, we evaluated our smart contracts by performing a sequence of transactions on a simulated Ethereum network, logging the gas consumption of each transaction.

The sequence of transactions is as follows:

1. Register the doctors and patients on the network.
2. Find out a doctor who has not created medical records for patients, perform the next step, and if such doctor does not exist, the procedure ends.
3. This doctor creates 10 medical records for each patient.
4. Each patient treats doctors as data viewers and authorizes every doctor to their complete medical records.
5. Transfer each patient’s complete medical records to every doctor.
6. Go to step 2.

In the first experiment, we also do a simple test to evaluate the performance of the search contract.

For the second experiment, we vary the number of mining nodes and measure transaction throughput and latency.

Through the first experiment, we can select the most optimal smart contract structures for this further performance evaluation. The objective of this experiment is to assess whether the smart contract structure can maintain an acceptable performance as the number of nodes in the blockchain network grows.

With the growth of the number of nodes in the blockchain network, we conduct user registration, creation of data, data update, viewer authorization, and data transmission, as well as recording transaction latency and system throughput.

### Experimental results

In the first experiment, we test six smart contract structures. First, Let’s look at the overall performance of these six smart contract structures. The total number of transactions and gas cost of each contract structure is summarised in Table 3. We execute various functions in smart contracts by sending transactions. So the number of transactions directly reflects the number of activities in these smart contracts. Due to some differences in the design of various smart contract structures, although the total number of transactions of the six smart contract structures are at the same level, there are some differences. It can be seen that PVR-centric contract structures have the lowest number of transactions because the PVR-centric contract structure is more suitable for many authorization and viewing operations. In addition, there is no need to support data update, so some transactions are omitted in the PVR-centric contract structure. On the other hand, the PVR-centric contract structures also have the lowest gas cost. The gas cost of the PPVR-centric contract structure is higher than that of PVR-centric contract structure. This is the cost of supporting data update. Obviously, both of these two structures we designed have a much lower cost than PPR-centric contract structures. This is because in PPR-centric contract structures, the viewer authorization and data transmission need to pay higher gas cost. We discuss the results further below, in terms of the key functions executed on the blockchain.

**Table 3:** Summary of results

Contract structure	Number of transactions	Total gas cost
PPR-centric	66010	26,500 million
PPR-centric,group-based access	69510	24,200 million
PVR-centric	64010	12,992 million
PVR-centric,group-based access	60010	12,800 million
PPVR-centric	64510	15,800 million
PPVR-centric,group-based access	64510	15,700 million

Gas consumption of creating medical records for patients is approximately constant with the number of medical records added, and implementing group-based access does not affect this trend. In both implementations of the PPR-centric contract structure, the gas cost of a single data creation operation fluctuates around an average of 209,000 while in the PVR-centric contract structures, the average gas cost is slightly higher at 230,000. In the PPVR-centric contract structures, the average is 274,000. This is likely because creating medical records only adds records to a smart contract that stores medical records, eliminating traversal across contracts. Group-based access is used to manage authorization and transfer data, not to create data. Such experimental results are expected.

**Fig. 5:**
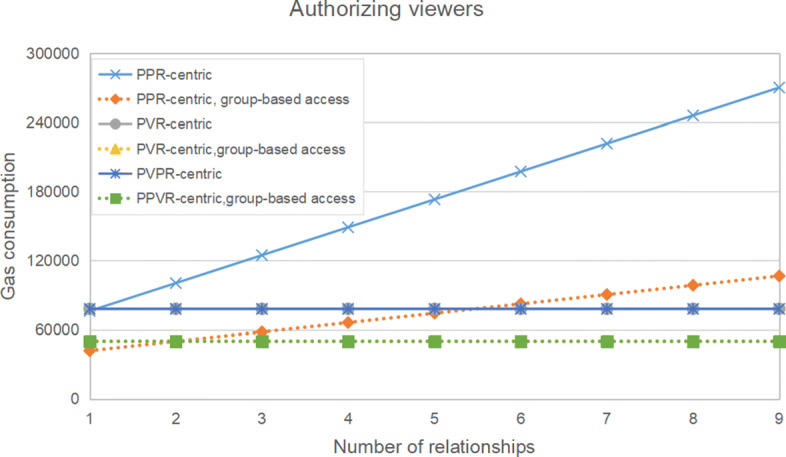
Gas consumption of viewer authorization

Table 4 and Figure 5 show the gas consumption of granting authorization to a viewer, against the number of doctors the patient has previously seen (i.e. the number of PPR). We can see that our hypotheses in Section 4 are verified. It is obvious from the figure that as the number of PPR increases, the gas consumption of authorizing a viewer increases linearly in the PPR-centric contract structures with or without group-based access. Although group-based access doesn’t change the trend, it lowers gas consumption significantly, as the authorization is simpler. Correspondingly, this remains constant in the PVR-centric contract structure and the PPVR-centric contract structure. Because the values are very close, the lines of the PVR-centric contract structure and the PPVR-centric contract structure overlap completely. Similarly, the lines of these two structures with group-based access also overlap. So, for more specific information about the PVR-centric contract structure and the PPVR-centric contract structure, we can refer to the Table 4. This overlap is also very easy to understand. The biggest difference between the PVR-centric contract structure and the PPVR-centric contract structure is that the PPVR-centric contract structure adds data reviewers and designs to support data update. As for the part of authorization, these two structures are almost the same. Therefore, whether implementing group-based access, it is natural that the gas consumption of these two smart contract structures is almost the same. On the whole, on the one hand, compared with the linear growth of the PPR-centric contract structures, the PVR-centric contract structures, and the PPVR-centric contract structures can keep a low constant. On the other hand, group-based access can significantly reduce the gas of these smart contract structures.

**Table 4:** Gas consumption of viewer authorization

Structure	Number of PPR								
	1	2	3	4	5	6	7	8	9
PPR-centric	76217	100472	124727	148982	173237	197492	221747	246002	270257
PPR-centric,group-based access	41923	50017	58111	66205	74299	82393	90487	98517	106675
PVR-centric	78109	78109	78109	78109	78109	78109	78109	78109	78109
PVR-centric,group-based access	50184	50184	50184	50184	50184	50184	50184	50184	50184
PPVR-centric	78045	78045	78045	78045	78045	78045	78045	78045	78045
PPVR-centric,group-based access	50072	50072	50072	50072	50072	50072	50072	50072	50072

**Fig. 6:**
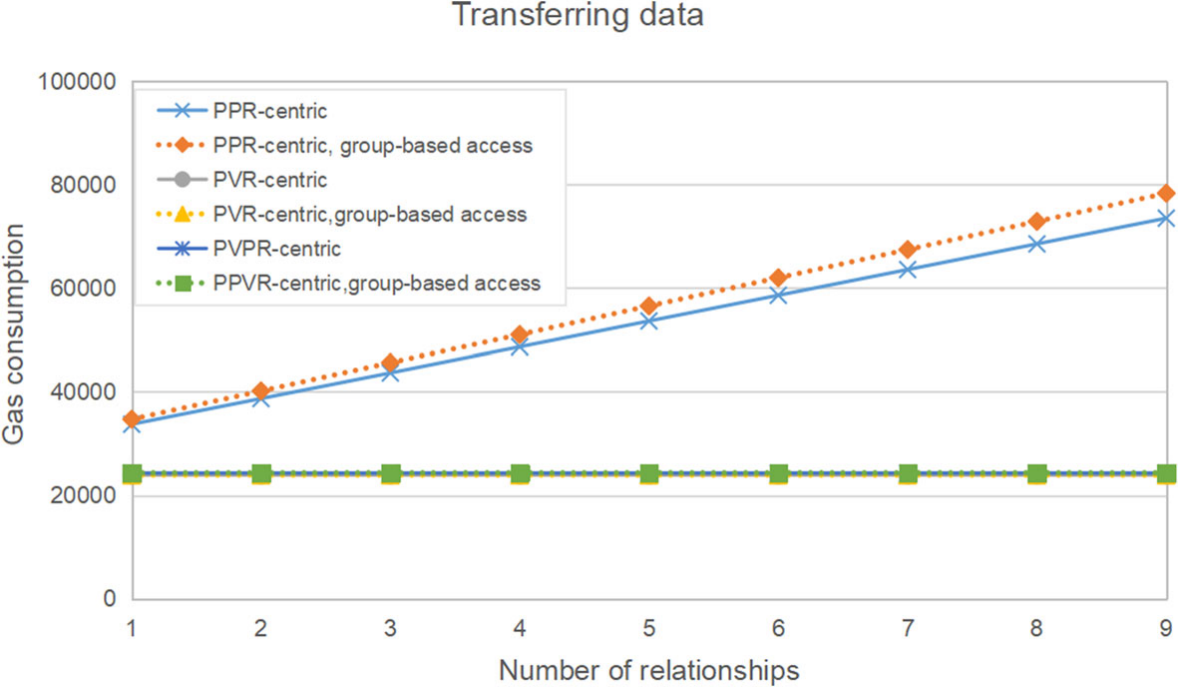
Gas consumption of data transmission

Table 5 and Figure 6 show the gas consumption of data transmission from a patient to a viewer, against the number of PPR. Similar to viewer authorization, from the figure, we can see that the gas consumption of transferring health data also increases linearly in the PPR-centric contract structure with or without group-based access. Compared with reducing gas consumption greatly in viewer authorization, group-based access only slightly reduces gas consumption of data transmission in the PPR-centric contract structures. In the PPR-centric contract structure, a patient’s health data is stored in multiple smart contracts. To transfer the complete health data, the system needs to traverse multiple smart contracts. Naturally, gas consumption increases with the growth of the number of doctors the patient has previously seen. Correspondingly, the gas consumption remains constant in the PVR-centric contract structure and the PPVR-centric contract structure. Because the PVR-centric contract structures and the PPVR-centric contract structures are basically the same in data transmission, and group-based access has little effect, the gas consumption lines of the four smart contract systems are overlapped. Specific data can be seen in the Table 5, the differences between these values are very small. In general, the trend of gas consumption is the same as that in viewer authorization. The difference is that group-based access has little effect here.

**Table 5:** Gas consumption of data transmission

Structure	Number of PPR								
	1	2	3	4	5	6	7	8	9
PPR-centric	33746	38715	43685	48655	53624	58594	63564	68534	73504
PPR-centric,group-based access	34728	40177	45627	51007	56525	61976	67426	72876	78326
PVR-centric	23933	23933	23933	23933	23933	23933	23933	23933	23933
PVR-centric,group-based access	23937	23937	23937	23937	23937	23937	23937	23937	23937
PPVR-centric	24300	24300	24300	24300	24300	24300	24300	24300	24300
PPVR-centric,group-based access	24355	24355	24355	24355	24355	24355	24355	24355	24355

In the simple test for our search contract, we find that updating the keyword list of a patient incurs an average gas cost of 63,000 per keyword, and a query incurs an average cost of 25,000. Since the minimum gas cost of a transaction is 21,000, we can see that our search contract operates efficiently, incurring relatively little gas to update keywords and execute queries.

From our results, we can see that the improvements proposed in Section 4.3 do indeed bring obvious benefits to these smart contract structures. On the one hand, group-based access lowers the gas consumption and thus computational complexity of executing the key functions of our framework. On the other hand, the search contract improves the practicability of our framework at a low cost. Most significantly, the design of the PVR-centric contract structure and the PPVR-centric contract structure allow our framework to scale sustainably as the network becomes denser and patients interact with more medical providers. In both viewer authorization and data transmission, the PVR-centric contract structure and the PPVR-centric contract structure improves performance from *O*(*n*) in the PPR-centric structure to *O*(1). This has reached optimal complexity. Therefore, it can be concluded that the PVR-centric contract structure and the PPVR-centric contract structure have better performance than the PPR-centric contract structure.

Because the PVR-centric contract structure with group-based access is the most optimal structure in terms of performance, we select it to conduct the second experiment to further evaluate its performance.

**Fig. 7:**
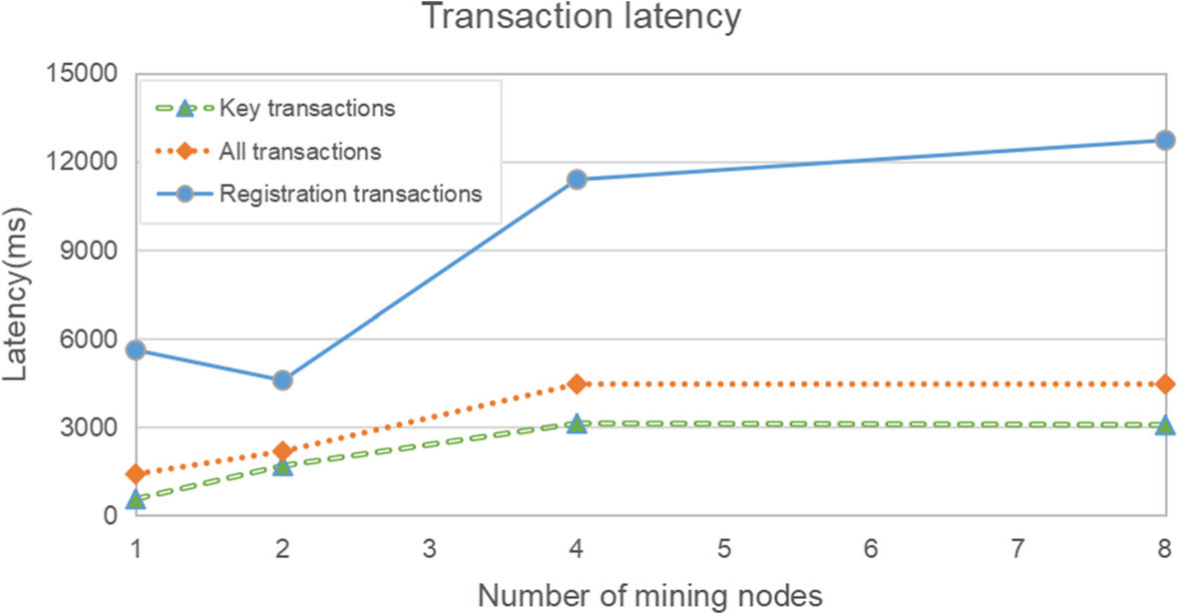
Transaction latency

Due to the limitation of experimental conditions, the experiment only simulate four different mining situations in which the maximum number of mining nodes is 8. Therefore, we can only simply find some trends and make reasonable conjectures. From Figure 7, it can be seen that transaction latency generally increases as the network size increases. As registration transactions are one-time actions, they are not as significant as data creation, viewer authorization and data retrieval transactions (the key transactions). Therefore, in order to evaluate the performance more representative, we observe the latency from three perspectives: All transactions, Key transactions and Registration transactions. For all transactions, the average transaction latency increases from about 2s with 1 node, to just over 4 s with 8 nodes. Although there is a constant sign from 4 nodes to 8 nodes, it is difficult to conclude that the latency will not continue to grow when there are more mining nodes. From the operation of Ethereum mainnet, when there are many mining nodes, the time required for a transaction to be packaged into a block will not be too long if the network is unobstructed. For registration transactions, the latency is significantly higher than the average. However, the frequency of registration transactions is relatively low, so such a high latency is acceptable. When considering the average latency of key transactions, latency is more acceptable, increasing from less than 1s with 1 node to about 3s with 8 nodes.

**Fig. 8:**
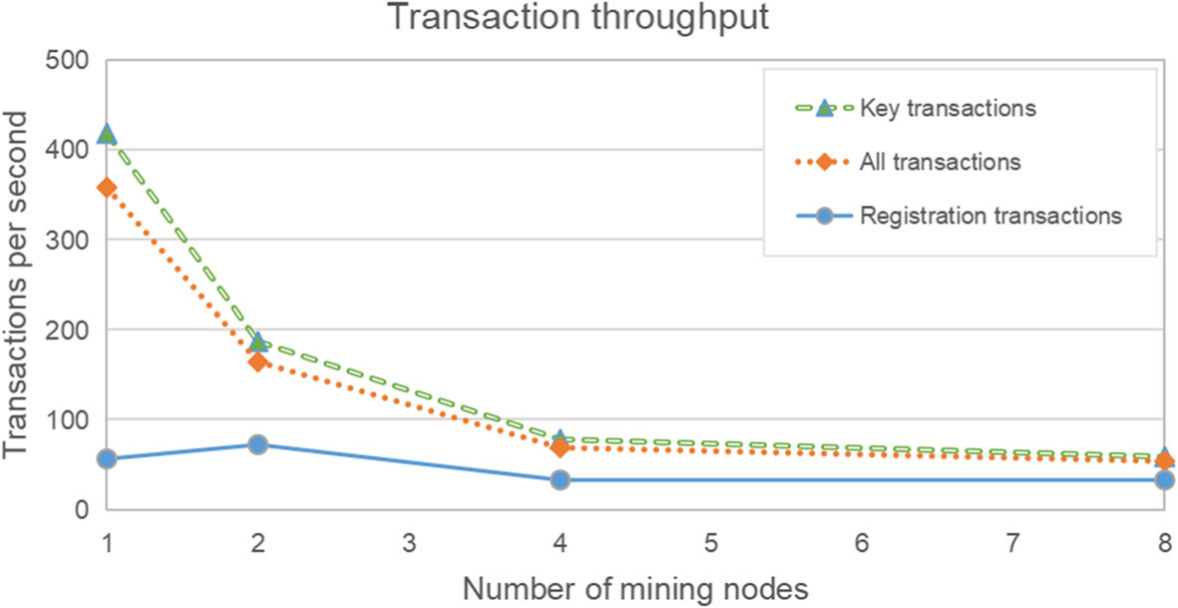
Transaction throughput

Figure 8 shows that transaction throughput falls as the network size increases. This is expected as transaction latency increases with network size; as transactions take longer to be confirmed, the number of transactions confirmed per second falls. Average throughput falls from about 350 transactions per second with 1 node, to just over 50 with 8 nodes. Again, the throughput for registration transactions is significantly lower than average. When only key transactions are considered, throughput is about 425 transactions per second with 1 node, to about 50 with 8 nodes. Such a low throughput is unacceptable, but it should be noted that this is limited by the experimental environment, so it can only reflect a simple trend. With the increase of the number of mining nodes, the transaction throughput may decline. Therefore, if we limit the number of mining nodes and use high-performance physical machines, the throughput should reach a high level, which needs a further experiment.

Taken together, the significantly higher latency and lower throughput associated with registration transactions are due to the higher gas costs they incur. In Ethereum, when there are enough transactions in the transaction pool, it is the GasLimit allowed by the block that limits the number of transactions in that block. Fewer registration transactions can be mined per block, resulting in higher latency and lower throughput. The importance of keeping gas consumption low in smart contract design can thus be seen in the performance impact of high-gas transactions.

## Discussion

In this section, we first analyze our findings. Then we describe our prototype and discuss the potential application scenarios of such a system in the current COVID-19 outbreak. Finally, we deliberate the limitations of our research.

As shown in the previous section, the performance of the blockchain deteriorates as the network size increases. Consensus takes more time, which increases transaction latency and reduces throughput. This harms the system as a whole. Patients have to wait longer to confirm their trades, which makes it difficult for them to track their data accurately. Nevertheless, even with poor latency and throughput, CrowdMed-II can continue to run reliably, despite a significant deterioration in the user experience. The low performance of CrowdMed-II can be alleviated by using a consortium blockchain, in which nodes must be approved to join the network. This limits the number of nodes in the blockchain to ensure acceptable performance.

The inclusion of data reviewers in the CrowdMed-II framework allows data reviewers to review patients’ medical records, evaluate treatment protocols of medical service providers, and correct and supplement keywords of patient data, which not only improves the quality of health data but also adds additional knowledge to it and allows viewers to search and obtain the desired data more quickly and accurately. With the participation of data reviewers, the system can operate and develop more healthily.

The smart contracts in this paper support the implementation of a blockchain-based framework for health data sharing. The smart contract structures we designed, along with group-based access rights, ensures the scalability of the framework as the number of users increases. It can be observed that the cost of the gas for critical functions has not increased rapidly as patients interact with more providers, which is critical to the goals of our framework. Both PVR-centric contract structure and PPVR-centric contract structure have their own advantages and disadvantages. The former can not modify the data and is more friendly to the data viewer because the data used can be obtained repeatedly without modification. But it also takes up more storage space and requires data creators to make few mistakes. The latter allows data creators to update data, which is conducive to maintaining the enthusiasm of data creators and saving the storage space of the blockchain. Correspondingly, the data viewer needs to store the data used locally.

Including a mechanism that allows medical researchers to search for patients also enhances the practicability of our framework. Researchers can search for highly specific patient data and creating health datasets that contain large amounts of useful data to gain insight. For example, a study of heart disease patients can search for and obtain medical records from patients with heart disease.

By empowering patients in the sharing of health data, patients are able to access their own complete health data and have free access to the provider of their choice. Because health data can be shared over the Internet, patients can also receive medical advice remotely from online medical service providers, which would be beneficial in the current outbreak of COVID-19. In such cases, patients may interact with more medical service providers than they currently do. They are free to seek a second, third or even fourth opinion on the medical diagnosis and prescribed treatment plan. Empowering patients will democratize the medical industry because patients have greater bargaining power.

If CrowdMed-II is adopted globally, the data collected can create a comprehensive dataset across countries, age groups and backgrounds of patients, from which new insights can be gained. Using today’s traditional techniques and data collection methods, medical researchers find it very difficult to collect such data. In addition, medical research institutions, especially smaller ones that are not supported by larger organizations, will no longer be hampered by difficulties in accessing sensitive, real-world health data.

We develop a prototype system. At the registration interface, users can register different types of accounts. When patients log in to the system, they can set different access rights for different groups and set the group of viewers. They can also add labels (i.e. keywords) to their data for searching contracts. Patients also have access to all their health data. When medical service providers log in to the system, they can not only upload the patient’s health data but also add labels to the patient’s health data. When viewers log in to the system, they can use the search function supported by the search contract to find the patients who meet their query conditions and then use the query results to retrieve the corresponding data.

In the current outbreak of COVID-19, this system can play a very positive role. If it is adopted globally, then when the coronavirus mutates in one place, medical institutions and medical researchers all over the world can obtain detailed data at the first time. This can prevent the further spread of mutated coronavirus in the world to the greatest extent. In addition, the system can also play a role in the sharing of health data such as health QR code. In China, each province has its own health QR code, and the large-scale homecoming during the Spring Festival has brought challenges to the sharing of health QR code. If the health QR code is connected to our system, the sharing problem can be effectively solved.

Although we have done a lot of work, several areas of the implementation of our framework are not discussed in depth in this article. Firstly, the construction of the query string (especially the string describing access rights) is simplified in this paper. Constructed query strings should allow access to the required subset of medical records, which can be achieved by borrowing techniques from a great deal of research on string similarity searches and join [34–36, 38–41]. Moreover, the patient should be able to select fields and spans that viewers can access. This can complicate the construction of the query string. However, this would be handled by the application rather than by the blockchain. So we didn’t take that into account when we developed the smart contracts. Secondly, how the query string will be executed on the provider’s database is also simplified here. We assume that a URL endpoint would handle this, and that minimal additional information would need to be encoded in the smart contracts. Thirdly, it is very difficult to screen patient data only by keywords. In actual medical research projects, the screening conditions of patient data will be very complex. Inspired by [43], we can transform health data into vectors through representation learning, and then search and match in vector form. Lastly, the limitations of blockchain technology remain a potential obstacle to our framework. With continuous improvement of blockchain technology, we hope that the blockchain system and network can overcome the current problems of throughput and latency, such as those dealing with streaming data [4, 13, 16]. In emergency situations, health data is often urgently needed. Therefore, the practical applicability of our framework depends on the continuous improvement of the Ethereum network and the blockchain.

## Conclusion and future work

We propose a framework that empowers patients with their health data, encourages more sharing of health data and brings in data reviewers to improve the quality of data. The smart contracts developed in this paper effectively perform the service functions of the framework. As our experiments show, our optimal design scales linearly with network density and have been significantly improved in previous work in this field. In addition, the introduction of group-based access rights and the search contract has greatly increased the practicability of our framework. We want smart contract developers to be able to adapt our contracts for applications in other areas, especially in the Internet of Things where data access management is a key issue.

In the future, we intend to create a complete, working framework to address the limitations described in Section 6 of this paper. In developing the framework, ensuring that the application is secure, protecting patient privacy is our main priority, and that design is intuitive and useful. But at present, because real data is stored in the databases of medical service providers and the blockchain stores only a query string and a data hash, which can only guarantee that data tampering will be discovered and not that it will not be tampered with, we can further study in this direction. Finally, data harmonization remains an obstacle to the integration of data from different sources, particularly in the case of health data for which there is no well-implemented common data standards. We intend to explore this subject further and to integrate existing processes into our framework in order to fully realize the future of patients having full ownership of their health data and the ease with which they can be shared and mined for valuable insights.
